# Experiences of Living With the Nonmotor Symptoms of Parkinson's Disease: A Photovoice Study

**DOI:** 10.1111/hex.14124

**Published:** 2024-06-25

**Authors:** Laura J. Smith, Jerri Callis, Shannon Bridger‐Smart, Olivia Guilfoyle

**Affiliations:** ^1^ Preventive Neurology Unit, Wolfson Institute of Population Health Queen Mary University of London London UK; ^2^ School of Psychology, Keynes College University of Kent Canterbury UK; ^3^ Salomons Institute for Applied Psychology Canterbury Christ Church University Tunbridge Wells UK

**Keywords:** activities of daily living, nonmotor symptoms, Parkinson's disease, participatory action, photovoice, quality of life

## Abstract

**Background:**

Nonmotor symptoms (NMSs) are frequently experienced by people with Parkinson's disease (PD) and are often perceived as their most bothersome symptoms. However, these remain poorly understood with suboptimal clinical management. These unmet needs are an important determinant of health‐related quality of life (QoL) in PD.

**Objective:**

The aim of this study was to gain insights into the experience of living with the NMS of PD in real‐time using participatory action methodology.

**Method:**

Using the photovoice method, 14 people with PD took photographs to document their experiences of living with the NMS of PD. They composed corresponding written narratives to capture the impact of NMS on their daily activities and QoL. In total, 152 photographs and corresponding narratives were analysed using thematic analysis with an inductive approach.

**Results:**

Four interrelated themes were identified. Emotional well‐being and sense of self encompassed a process of adjustment to living with PD. Engaging in valued activities, adopting a positive mindset and utilising coping strategies were thought to enhance confidence and self‐esteem. Social support and societal awareness highlighted the importance of supportive relationships and socialising to aid participation and avoid isolation. Barriers to social engagement included the unpredictability of NMS and nonvisible NMS being neglected or misunderstood.

**Conclusion:**

Findings demonstrated the far‐reaching impact of nonmotor aspects of PD on emotional, occupational and social dimensions. These needs could be addressed through person‐centred and comprehensive approaches to care.

**Patient or Public Contribution:**

This study utilised a participatory research approach allowing participants to choose the subjects that mattered to them and how to present their results. Additionally, a group workshop was held with people with PD, their family members and healthcare professionals to guide theme development.

## 1 Introduction

Parkinson's disease (PD) is the fastest‐growing neurological condition worldwide [1]; 145,000 people in the United Kingdom are currently estimated to be living with PD [2]. PD is an incurable neurodegenerative disease, resulting from the progressive loss of dopamine‐producing neurons in the substantia nigra. PD was traditionally characterised as a movement disorder with four key symptoms: bradykinesia, rigidity, postural instability and tremor [3]. Nonmotor symptoms (NMS) are also commonly experienced by people with PD. They can precede motor symptoms by five or more years and are the presenting clinical feature of PD in over 20% of individuals [4]. These include cognitive dysfunction, mental health disturbances, sleep disorders, anosmia and ageusia, and bladder and bowel dysfunction [3].

NMS are often listed as the most bothersome symptoms by PD patients [5], and have been demonstrated to have a greater impact on health‐related quality of life (QoL) compared to motor symptoms [6]. NMS are also an important determinant of loss of independence affecting decisions around residential placement and compounding caregiver burden [7]. Considering the impact that NMS holds on persons with PD, identification and treatment of these is essential to promote well‐being.

Currently, the management of NMS remains suboptimal. The reasons for this are multiple and include reduced awareness and an insufficient evidence base for effective treatments [8]. Pressures on healthcare services also contribute to unmet needs. Access to a Parkinson's nurse is often limited, the healthcare professional with whom patients are most likely to discuss NMS [9]. Provision of nonpharmacological therapies (including occupational therapy, speech and language therapy and counselling) also varies across the United Kingdom and referral times are generally delayed [2]. Taken together, these factors often result in people with PD feeling unsupported [10, 11] and raise questions about how patients are managing the impact of NMS in their daily lives.

NMS can affect one's daily functioning and QoL in various ways, though these symptoms may not be visible. The way in which an individual copes with these symptoms is personal [12] and influenced by contextual and sociodemographic factors [13]. Recent data indicate that coastal communities experience health inequalities, with some of the worst health outcomes and lowest life expectancy in England [14]. While those living in rural communities face challenges accessing healthcare specialists nearby [15]. Currently, there is a lack of research focusing on the lived experiences and daily life challenges faced by individuals with PD and how NMS contributes to this. To better understand this, a community‐based, patient‐centred approach is needed to inform local strategies that best meet patients' needs [16]. Kent in Southeast England is characterised by degrees of health inequality primarily due to ‘coastal excess’ across its numerous coastal and rural communities [17]. Therefore, we aimed to leverage our unique location to explore the challenges faced by people with PD in these communities.

Photovoice is a qualitative research method increasingly used in health research to understand illness experiences from the patient's perspective [18]. The approach involves participants taking photographs that illustrate their circumstances relating to a research topic. The tool promotes reflection and empowerment amongst participants, while researchers can gain unique insights into individuals' lives [19]. To date, four PD photovoice studies have been conducted. Roger, Wetzel and Penner [20] conducted a case study to explore experiences of invisibility in PD. Strong spousal support helped overcome invisibility in social contexts and defined the individual's experience of living with PD. Hermanns, Greer and Cooper [21] explored the everyday challenges of PD with nine participants, revealing a variety of coping strategies to manage symptoms and stay determined. Similarly, Lutz et al. [22] invited six people with PD to share how they navigate living with PD, highlighting a strong connection between occupation and sense of identity. Finally, Greer, Hermanns and Cooper [23] investigated nine participants' experiences of aging with PD, identifying determinism to live with the disease and overcome losses experienced.

No studies have applied photovoice to specifically explore NMS in PD. Since NMS negatively impact QoL, are underrecognized and often less visible, further insights are required. Therefore, the aim of our study was to explore the lived experiences of people with PD living across rural and coastal communities in Kent, specifically in relation to their NMS, using photographs and narratives. This could raise awareness of the impact of NMS and help identify topics that may benefit from ongoing advocacy.

## 2 Method

### 2.1 Design

This is an explorative, qualitative study with a photovoice design. Photovoice is a participatory research method whereby participants create their own photography and accompanying narratives to capture their subjective experiences [24]. Photovoice was employed to offer new insights into the NMS of PD since it aligns with a person‐centred approach allowing participants to focus on what matters to them. The method also allows participants to share their experiences in a real‐time manner rather than asking about these retrospectively [25].

This study follows the steps for conducting photovoice studies outlined by Wang and Burris [24] and guidelines from Sutton–Brown, which illustrate how to implement these steps in different contexts to meet the needs of individuals taking part [26]. Findings are reported based on the SRQR Reporting Checklist for Qualitative Data [27]. We applied Yardley's [28] evaluative characteristics, which outline flexible principles to guide the quality of qualitative methods in health psychology.

The authors conducting this research consisted of: L.J.S., a lecturer in health psychology, J.C. a clinical doctoral student and S.B.‐S. and O.G. who both hold an MSc in cognitive neuropsychology.

### 2.2 Participants

The typical sample size for photovoice methodology is 7–10 participants [18]; we therefore determined that a sample size of 10–12 participants would be sufficient. The research team regularly reviewed and discussed the data to determine whether data saturation had been met. Saturation was operationalised as the point where no new data were generated that supported ongoing theme development.

This study took place in Kent, England, where 3760 people are estimated to be living with PD. Ninety‐eight percent of these are White, 69% are aged 65–79 and 1180 live in rural areas [29]. Using a convenience sampling approach, participants were recruited through local Patient and Public Involvement (PPI) networks, Parkinson's UK Research Support Network and word of mouth. Inclusion criteria were having a PD diagnosis, residing in the United Kingdom, >18 years of age and being able to communicate in English. Seventeen participants signed up for the study; however, three dropped out due to personal commitments or illness. Fourteen community‐dwelling participants completed the study. Fifty percent of the sample were female and all identified as white British. Ages ranged from 45 to 80 (*M* = 65.43, SD = 8.69). On average participants were diagnosed 5.58 years ago (SD = 4.11). See Table 1 for additional demographic information.

**Table 1:** Participant demographics.

Gender	Age	Ethnicity	Education level	Employment	Years since diagnosis
Female	45^[Link]^, ^a^	White	Degree	Self‐employed PT	0.83
Female	67	White	Other	Retired	7.42
Male	60	White	A level	Medically retired	11.58
Female	59	White	GCSE	Employed FT	6.58
Male	63	White	GCSE	Medically retired	1.75
Female	65	White	Degree	Employed FT	3
Female	61	White	A level	Medically retired	11
Female	67	White	Other	Employed PT	6
Male	69	White	GCSE	Medically retired	13.92
Male	66	White	Degree	Retired	5.58
Male	80	White	Other	Retired	2.33
Male	77	White	GCSE	Retired	2
Female	75	White	Other	Retired	3.25
Male	62	White	Degree	Employed PT	2.83

Abbreviations: FT = full‐time; PT = part‐time.

^a^ Diagnosed with young onset PD. Participant demographics are presented in random order and without identifiers to protect participants' identities.

### 2.3 Materials and Procedures

Following informed consent, participants attended an individual training session with a researcher in person or via videocall. During the session they were talked through a training booklet, which provided information on the study rationale, how and what to take photographs of and what content to include in their narratives.

Participants were asked to take photographs to document aspects of their daily lives that relate to the NMS of PD and write an accompanying narrative for each photograph to explain its meaning. This included places, objects or events that either enhance or challenge their daily activities and QoL. They were invited to share both the negative and positive aspects of living with PD.

A template was provided to help participants develop their accompanying narratives (see Files S1). The template provided prompts for participants to write their reflections on what was happening in the photograph, why they took the photograph, what it meant to them and how the content related to their experiences of NMS. Template questions were adapted from the SHOWeD technique outlined by Wang and Burris [24] and Wang et al. [30] and previous photovoice literature [22, 25]. Participants could complete a paper‐based or digital version of the template. Completion could be supported by a family member or using dictation tools.

One participant used a disposable camera (provided by the researchers), and the remainder used their own devices. They were asked to take 10–15 photographs over a 2‐week period [26]. Most met this deadline, but two participants required 3 weeks. Participants sent their photographs and reflective templates to the research team via secure email, electronic transfer or post. A £20 shopping voucher was given to each participant as a thank you for taking part.

### 2.4 Ethical Considerations

Ethical approval was granted by the University of Kent School of Psychology Ethics Committee (reference: 202216473327287620). All participants provided informed consent before data collection. A release form was obtained from individuals who appeared in participants' photographs.

### 2.5 Data Analysis

We followed photovoice procedures developed by Wang and Burris [24, 31], which prioritise interpretation of the photographs, rather than the photographs themselves. Narrative data taken from the reflective template for each photograph were analysed using thematic analysis, following the approach outlined by Braun and Clarke [32, 33]. An inductive approach with an essentialist/realist epistemology was chosen, which assumes a simple unidirectional relationship between the meanings and realities within participants' data, since our aim was to understand how photographs were interpreted by participants [33]. Photographs were analysed alongside the narratives to understand the meaning that participants had assigned to these, as opposed to overlaying our own interpretations. The photographs enabled us to visualise and grasp participants' interpretations and explore the broader meaning of themes [19].

The authors first familiarised themselves with the data by collating, reading and making initial notes on their observations of the topics expressed in the data. The researchers individually coded a subset of the data (three different participants each) creating an initial set of meaningful components (codes) relating to the impact of NMS. They then met to discuss and compare ideas about which components were reoccurring or emphasised. Components were selected and grouped to develop a formal coding scheme (see Supporting Information: Materials) to apply to the remainder of the data set. As the analysis was data‐driven, further codes were added in response to emerging concepts.

After each participant's data had been double‐coded using the coding scheme, the researchers met to discuss how pertinent codes could be collated and categorised to establish initial themes. Photographs helped to unfold theme development at this stage. For validation purposes, these initial themes were presented to a PPI group consisting of people with Parkinson's who took part in the study, their family members and healthcare professionals. Photographs and quotes were displayed under each theme on poster boards for attendees to comment on. Initial definitions and interpretations of the themes were also shared via a slideshow presentation. Attendees provided feedback on whether the themes were representative and relatable and shared their interpretations. Learnings from this event informed the next step of theme definition and labelling; two researchers (L.J.S., and J.C.) collaboratively restructured the themes to align with the stakeholder discussions.

## 3 Results

Participants took 152 photographs in total, with an average of 11 per person. Four themes were extracted from the data: Emotional well‐being, Maintaining a sense of self, Support from others, and Societal awareness. These are described below with illustrative photographs and excerpts.

### 3.1 Emotional Well‐Being

**Figure 1:**
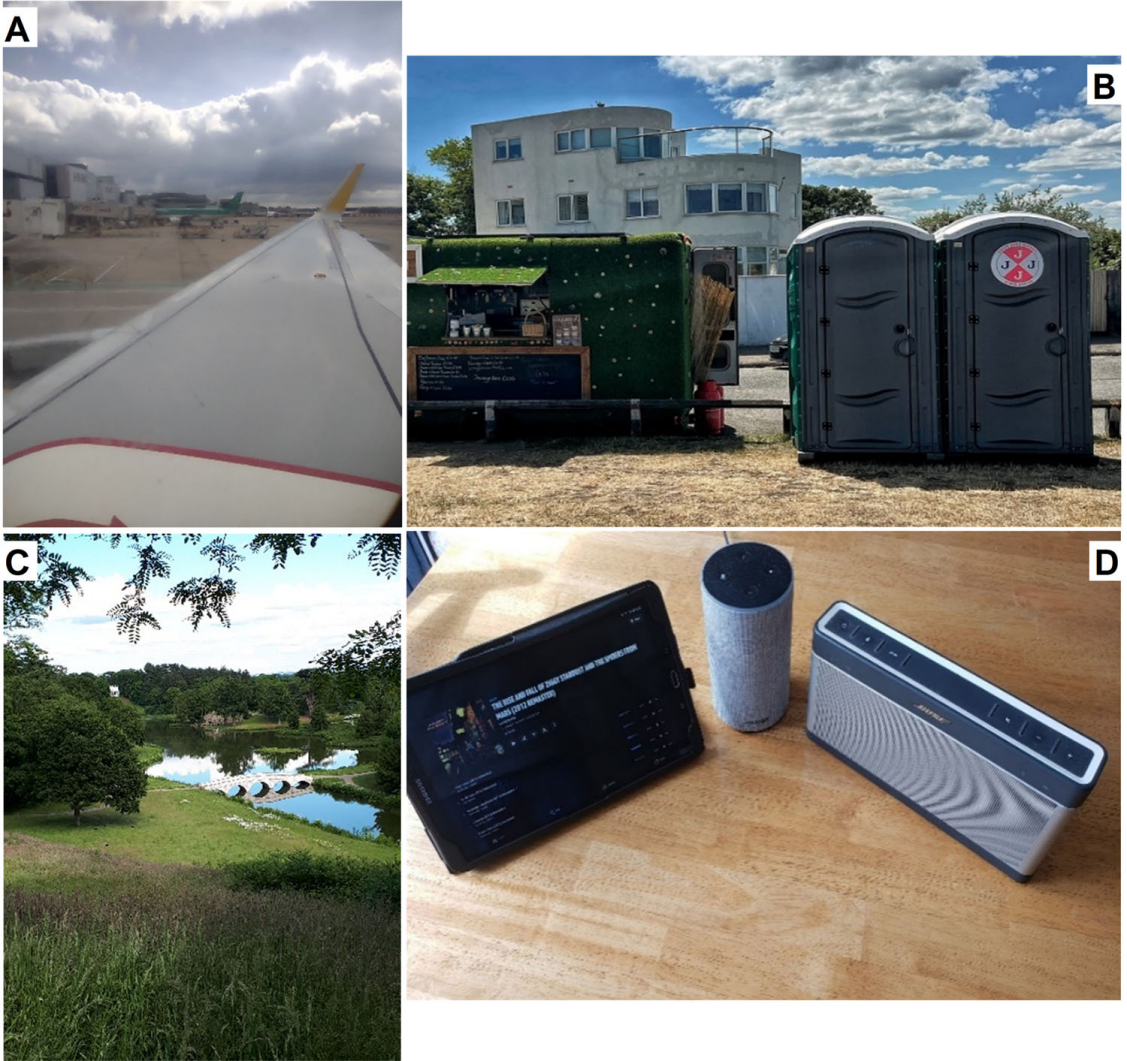
(A) Travel and holidays: ‘Lots of things now make me anxious, crowds, noise, confined spaces anything I can't control or get away from’. (B) Public toilets: ‘Being worried about finding a loo only adds to the anxiety which PD causes’. (C) Countryside: ‘It's positive to be able to spend time outdoors’. (D) Music: ‘Many Parkinson's people get depressed … and music can be a helpful remedy’.

Participants described changes to their mental health, which were perceived as both a NMS symptom of PD and an adjustment to the broader impact of living with PD (see Figure 1A). Anxiety, sadness, embarrassment, frustration and apathy were all frequently experienced and impacted upon QoL.

It might not be widely known but anxiety is common in people with Parkinson's. It's one of my worst symptoms and it's difficult to manage … It definitely limits what I feel comfortable doing.

(Participant 10)

Participants described how their mental health could interact with other PD symptoms to challenge activities of daily living (see Figure 1B). Mental health disturbances appeared to reduce confidence around undertaking activities, increase worrisome thoughts (‘what ifs’) and in some cases led to avoidant behaviours or social withdrawal.

Having this urgency [incontinence] is very embarrassing and governs my life. I can feel very uncomfortable and apprehensive about going somewhere, because I worry about the facilities. It is miserable, always needing the toilet, and the embarrassment and worry of having an accident.

(Participant 14)

Participants described actively changing their mindset to cope and come to terms with the long‐term emotional impact of PD. This encompassed adopting a positive and determined attitude to life and being driven towards achievable goals.

Concentrate on what you can do, not what you can't. It also helps with a positive mindset, which enables you to enjoy life more despite the difficulties Parkinson's bestows on you.

(Participant 9)

Others described learning how to manage patterns of negative thoughts, feelings and behaviours to achieve a meaningful life. Participants acknowledged this takes time and requires ongoing effort.

I experience periods of anxiety and low mood. I am learning to manage these thoughts and feelings with Cognitive Behavioural Therapy. Anxious thoughts can be all consuming and will often stop me from enjoying the fun things in life.

(Participant 7)

Participants also discussed the strategies they used to cope with and manage their mental health (see Figure 1C,D). Strategies included exercise, gardening, music, mindfulness, socialising and spending time in nature. Participants who were further along in their Parkinson's journey tended to embed these into their routine, which they felt benefitted both their physical and mental well‐being.

After a 45‐minute session exercising, I feel invigorated and ready to tackle the day ahead. It is good for both the body and mind, and I believe is very important for anyone who has Parkinson's.

(Participant 9)

### 3.2 Maintaining a Sense of Self

Participants highlighted the importance of preserving their identity. Engaging in valued activities that they had performed before the onset of PD helped them maintain a sense of normality and feel aligned with their former self.

I have been a runner since the age of eleven … long distance running gives me a feeling of accomplishment and energy. It allows me to still feel like ‘me’ without my PD! 

(Participant 7)

**Figure 2:**
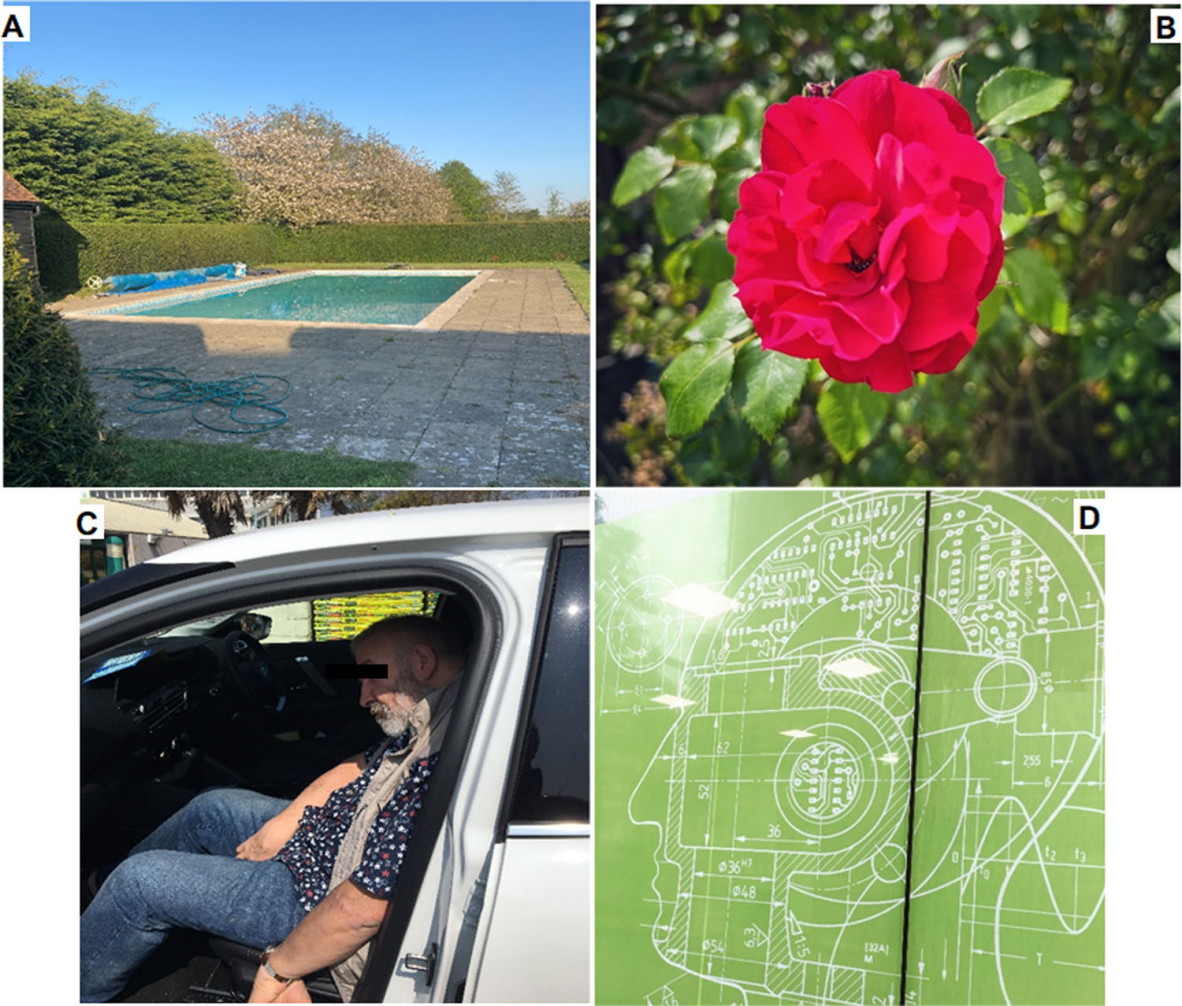
(A) Keep swimming: ‘It has always been an important form of exercise and I find swimming both relaxing and invigorating. Being able to swim is important to my confidence and morale’. (B) Missing the garden: ‘It makes me sad that I can't enjoy our garden as much as I used to’. (C) Driving and independence: ‘I miss doing something I previously enjoyed and not being able to drive is also a further blow to my independence’. (D) Proactive approach: ‘Gaining as much clinical information, being involved with research trials, working out what is best for my body and brain, is incredibly valuable and gives me purpose’.

Performing valued activities was described as having a positive impact on self‐esteem (see Figure 2A). Participants reflected on the sense of achievement gained by undertaking these and overcoming the hurdles presented by PD. This is turn enhanced their self‐worth.

It is an activity [painting] that I can enjoy despite having Parkinson's. Maintaining a sense of self‐worth helps with every other thing you do. It gives a sense of purpose and achievement.

(Participant 2)

In contrast, no longer being able to perform valued activities left participants with a sense of grief and loss (see Figure 2B), impacting their ability to fulfil social roles within relationships with family and friends.

I would often cook for friends and family. The loss of the senses of smell and taste has impacted on my ability to prepare meals and the enjoyment of eating. I feel this is another part of ‘who I am’ that has been taken from me.

(Participant 1)

Independence was considered an important factor for maintaining sense of self (see Figure 2C). Some participants felt dependent on others, affecting their autonomy, dignity, and self‐esteem: ‘I want privacy and to be able to do it on my own [using the toilet]’ (Participant 5). Participants wanted to retain a sense of freedom to engage in activities when, and how they wanted, without needing to rely on others.

Parkinson's makes you dependent, to some extent, on other people. Being able to go places under your own steam is very liberating and good for morale.

(Participant 2)

Participants expressed concerns about ‘the future and the progression of this disease’ (Participant 11) and therefore felt it was important to preserve their health to live well and retain a sense of self. Several had taken a proactive approach by developing their understanding of PD and making positive lifestyle changes (see Figure 2D). This helped participants to regain a sense of control over their NMS and feel more positive about the future.

Being able to research the latest developments related to Parkinson's gives me a sense of a degree of autonomy in managing my condition.

(Participant 2)

### 3.3 Support From Friends and Family

NMS affected participants' ability to undertake household chores, employment and outings. This impacted quality time spent with family and sometimes meant shifting responsibilities within the family unit to meet financial, domestic and emotional demands. Participants also described changes in their relationship dynamics, with some feeling disconnected or isolated from loved ones:

Due to my very disturbed nights, my wife and I no longer sleep together. It makes me sad that we no longer sleep together as I believe this is necessary to maintain a good marital relationship.

(Participant 1)

**Figure 3:**
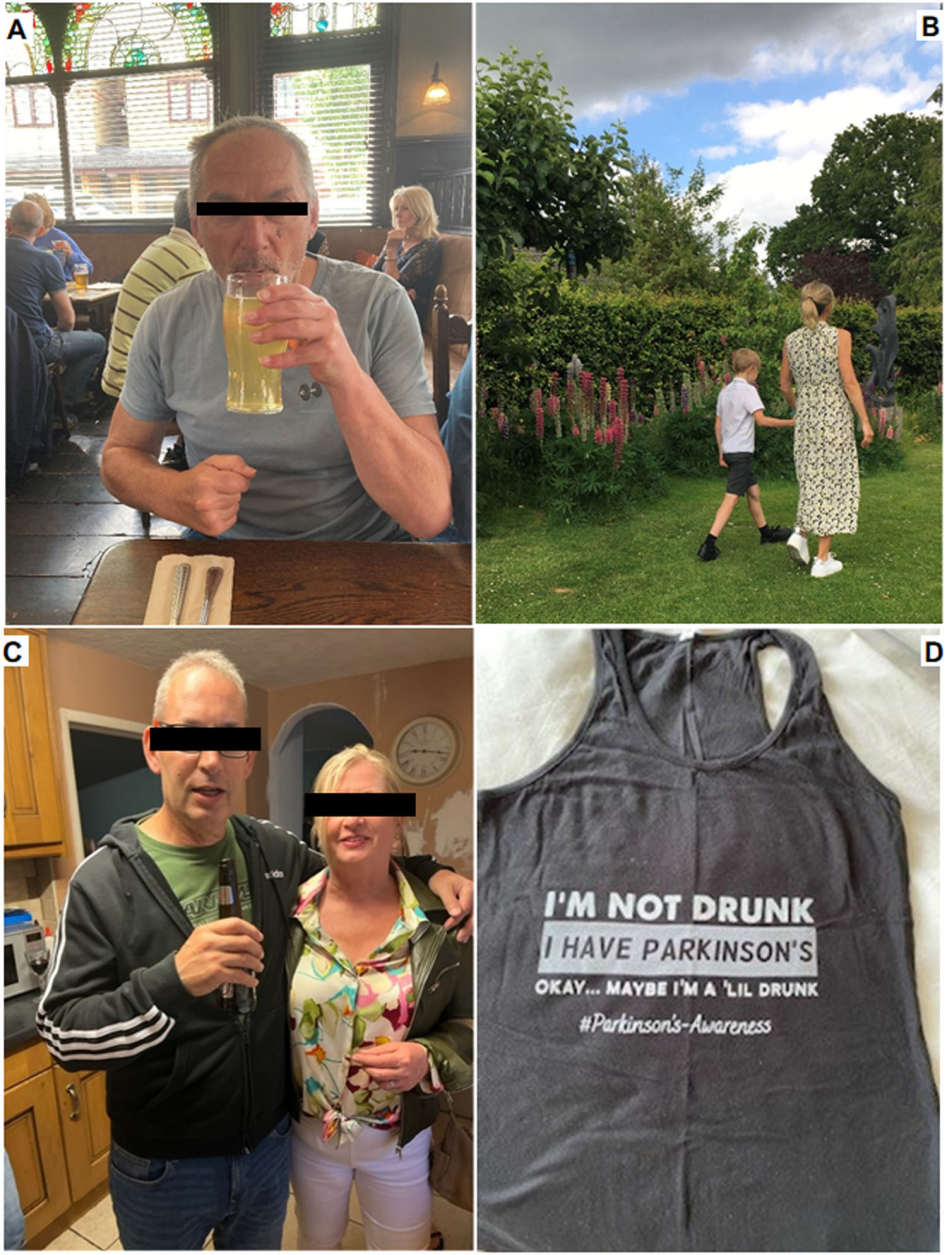
(A) Family support: ‘Being able to participate in social events is incredibly important for enjoyment and well‐being. I am lucky to have family around me who can assist me’. (B) Impact on family: ‘This disease isn't just about me. It will impact on my husband and our two boys. I worry about being a burden to them’. (C) Social isolation: ‘Turning down invitations to social gatherings in the fear that you might embarrass yourself puts restrictions on the way you live your life. Rather than playing safe and staying at home, give it a go’. (D) Public perceptions: ‘I don't want people to think I've been drinking when I haven't. It can make me self‐conscious when I cross a room’.

Participants were grateful for the support received from friends and family, which helped them to participate in daily activities but also maintain a positive mindset (see Figure 3A).

I am very lucky as I have a very supportive family and I think I would feel quite frightened sometimes if I had to face these problems alone.

(Participant 8)

However, participants did express concerns about burdening their loved ones and acknowledged the strain that NMS placed on relationships (see Figure 3B). Some concealed aspects of their PD from family to ‘show them I can manage’ (Participant 8) and try to alleviate the demands placed on loved ones.

This is so wrong, it is emotional dumping, she has never once complained, but I know I have to somehow stop it, as it is my disease, and the buck should stop with me … My plan is, instead of burdening my loved ones, I will write down all my worst thoughts and fears moans and tears in a private notebook which will then be closed and put away, hidden from sight.

(Participant 11)

Others described the challenges NMS presented in social situations. Invisible and unpredictable NMS which could make an ‘unwanted appearance very suddenly, with little or no warning’ (Participant 9) contributed to this. These experiences could induce loneliness or social withdrawal.

I find it hard to hold a conversation without losing the thread midway … Although I listen to the information being given to me, I find I forget it almost immediately and then ask silly questions. It makes social aspects very difficult.

(Participant 14)

Despite these challenges, participants felt it was important to engage in social activities to overcome feelings of isolation (see Figure 3C) and explained how they coped with this: ‘By looking at the funny side and self‐mocking, it should alleviate any awkward moments’ (Participant 9). They described how the support of friends and family can enable people with PD to engage in meaningful social activities.

Participation in social events is incredibly important for morale and self‐esteem … Encouraging people with PD and similar conditions to participate in social events is important and this can be helped by consideration of their particular issues, choice of venues, location, and time of day.

(Participant 2)

### 3.4 Societal Awareness

Being out in public sometimes induced embarrassment and anxiety, due to concerns about being judged by others. Participants further into their Parkinson's journey explained how some NMS are difficult to observe from the outside and therefore can be neglected or misunderstood by the public (see Figure 3D).

Some days I feel like I'm wearing a diving suit. I trudge around, feeling heavy and clumsy, and walking into things. But my suit's invisible—no one can see it, so they probably think I'm drunk.

(Participant 10)

Some acknowledged the helpful response of the public who were quick to assist once they became aware of their problem: ‘People are generally very kind if they know you have a problem but are not always aware just by looking at you’ (Participant 8). Looking forward, participants wanted to improve understanding of the NMS of PD amongst wider society. Greater awareness and an empathetic response were thought to be helpful in alleviating anxiety and embarrassment experienced by people with PD, and in turn facilitate their participation.

I want to take part and enjoy the company of my family and friends. If people were more aware of this problem, they would perhaps give me more time to join in. Educating people that those with Parkinson's need more time when conversing.

(Participant 1)

## 4 Discussion

Photovoices were captured to understand how the NMS of PD impact participation in everyday activities and QoL. Participants' narratives and photographs revealed how NMS impacted their autonomy, self‐esteem, relationships and socialising. Adopting a positive mindset, engaging in valued activities, implementing coping strategies and social support were important factors in adjusting to life with PD and maintaining well‐being.

Participants described changes to their mental health encompassing anxiety, avoidance, frustration, sadness and grief. This aligns with previous research showing depression and anxiety are commonly experienced in PD, with an estimated prevalence of 38% and 31%, respectively [34, 35]. Participants detailed how their mental health interacted with other PD symptoms, impacting upon their confidence to undertake daily activities, and leading to avoidance of situations. Accordingly, quantitative studies have shown anxiety and depression are associated with lower QoL [36] and cognitive [37] and motor decline in PD [38]. The participants in our study were aware of changes in their mental health and related these to their PD. This contrasts with previous research showing poor levels of awareness amongst patients and their families who had not connected mental health disturbances to PD [39], and other studies where patients had not volunteered information about their mental health because of social embarrassment [40].

Our findings align with existing psychotherapy frameworks applied in PD. First, participants discussed experiencing grief and adjusting to life with PD by adopting a positive mindset and engaging in valued activities. This is in accordance with Acceptance and Commitment Therapy (ACT), which aims to instigate valued activities to improve acceptance of distressing thoughts, sensations and emotions and in turn drive behaviour change [41]. Our findings indicate ACT's focus on meaningful activities, along with the acceptance of distress as a realistic response to PD may prove an effective strategy to enhance mental health [42]. Further, high‐quality RCTs are required to establish the benefits of ACT in PD [43]. Our findings also reflect how an individual's relationship with the self (low self‐esteem) and society (experiencing or expecting negative reactions from others) contributes to psychological distress (anxiety, depression, stress). This relates to Compassion Focused Therapy (CFT) frameworks, which aim to reduce internalised stigma and psychological distress by developing feelings of compassion towards the self and others [44]. CFT approaches that utilise the parasympathetic nervous system, acknowledge suffering and embody self‐compassion to alleviate it [45] have demonstrated positive outcomes in PD [46]. However, psychological formulations must acknowledge the impact of ableism, and not place the responsibility of embodying compassion solely on individuals with PD [47].

Participants also shared the strategies they implemented to manage the emotional impacts of PD and live well with the condition. These included practical strategies such as exercise, spending time outdoors and enjoyable hobbies, as well as cognitive strategies including pacing, adopting a positive mindset and accessing CBT. Our findings align with previous qualitative investigations that identified a range of self‐management practices amongst people with PD [48]. They also expand upon previous qualitative work by showing how self‐management experiences vary across the disease course [49]. Those in the earlier stages of PD were identifying and trying out strategies, while those further along were establishing these into a routine. We did not assess the level of severity or progression of PD symptoms here but recommend this be integrated into future research to further explore the timing of various self‐management components [49].

Importantly, these strategies were felt to benefit both physical and mental well‐being and could therefore be explored as part of a comprehensive approach to person‐centred PD care. Healthcare professionals could support patients to become more active in their own care by signposting reliable information and community services [50, 51, 52]. Clinical (NMS severity, disease duration) and sociodemographic (education, religion, family income, social support) characteristics have been identified as determinants of self‐management behaviours in PD [53, 54]. The strategies identified here are likely reflective of our sample who were White, English speaking, living in rural or coastal communities, educated and had access to social support. Healthcare professionals should therefore consider clinical and sociodemographic factors within their assessments and tailor self‐management support towards these.

Challenges to identity and perceived social roles were important, lowering participants' confidence, independence and self‐esteem. Living with the NMS of PD affected participants' self‐image, with some commenting that they no longer felt like themselves. Parallels can be drawn from previous literature where people with PD experienced grief for the past encompassing the person they were and the activities they can no longer perform [22, 55, 56], and this is the first qualitative study to evidence this specifically in relation to PD NMS. The onset of a chronic illness has been proposed to present an attack on the self‐concept challenging an individual's previously held view that their life would generally be positive, progressive and productive [57]. Participants discussed strategies they used to manage changes in sense of self, including maintaining existing valued activities and adopting a proactive approach. Although participants acknowledged they often needed to adjust to their current abilities, these activities provided a sense of achievement. Our findings echo those of Lutz et al. [22] demonstrating a strong link between sense of self and the activities people with PD engage in, as occupations were initiated, maintained, adapted or lost, one's sense of self was also impacted [22]. Occupational therapy programmes should therefore support people with PD to perform valued activities through the provision of adaptations and coping strategies [58]. Focusing on the individual's skills and interests (e.g., asset‐based approach) could be a means to increase their capability, opportunity and motivation [59]. Although occupational therapy is recommended for PD [60, 61], further research from well‐designed trials is required to guide programme design and implementation [62, 63].

Social support from family, spouses and friends helped individuals to participate, adjust to the consequences of PD and maintain a positive outlook. Participants felt more comfortable socialising with close others who understood their condition and could support them. PD may therefore have reduced the networks with whom they interact [22, 64, 65]. Some had resigned to a more passive social role or withdrawn from social situations because of the difficulties posed by their NMS and the unpredictable nature of PD. Social support networks are important for maintaining QoL amongst people with PD; conversely, social isolation is associated with reduced QoL, lower global functioning and depression [65]. Participants felt it was important to continue engaging in social activities, and care plans should therefore encompass social opportunities. Establishing a medication routine and adopting a positive mindset are considered helpful in managing social situations with PD [66].

Some participants, particularly those further into their Parkinson's journey, felt misunderstood by the public, which limited their activities and social engagement. Similar to previous work, participants were concerned about being mislabelled as intoxicated or nervous [67], which induced stress and embarrassment and impacted their self‐esteem. Public awareness campaigns could increase understanding of PD, particularly underrecognized and stigmatised NMS including facial masking, mental health disturbances, anosmia, and bladder and bowel dysfunction [68, 69]. Since these NMS often precede the onset of other Parkinsonian features, increased societal awareness could also promote earlier identification and treatment of PD [70]. For these to be effective, campaigns need to be targeted, sufficiently resourced and draw upon a range of stakeholders including policy makers, health advocacy organisations and people with PD to provide the necessary scale and close the translation gap [71].

Participants were concerned about being a burden to their family and friends and acknowledged the strains PD placed on relationships. NMS severity is associated with caregiver burden in PD; for example, disinhibition and agitation are associated with greater emotional burden [72], and speech abnormalities limit effective communication [73]. Some participants told us they tried to conceal their symptoms and emotions to avoid burdening close others. Research on ‘concealment’ coping strategies in other chronic health conditions shows mixed health outcomes [74]. Concealing symptoms could help avoid being stigmatised by others; however, it can also lead to internalising distress therefore reducing well‐being and access to social and medical support. Further research could explore the motivations and outcomes related to concealment in PD. Results from this study indicate that family dynamics play an important role in the well‐being of people with PD, and care provision should therefore consider the family context [75]. Family‐system interventions designed for other chronic conditions could be adapted for use within PD [76, 77].

This study utilised the photovoice method to visualise the everyday impact of NMS in real‐time and from the perspective of people with PD. The photographs provide accessible visuals to stimulate discussions with diverse audiences and capture patient experiences of NMS, which might typically be overlooked [78]. Accompanying narratives also provided insight into the personal realities of the participants aligning with a person‐centred approach. Adopting a participatory research approach allowed participants to choose subjects that matter to them, how to present their results and guide theme development.

The photovoice method demands more effort from participants than typical qualitative interviews which could be seen as a limitation [19]. Three participants dropped out of the study, possibly reflecting these demands. Our sample size (*N* = 14) exceeds the typical sample size for this methodology [18] and was varied in terms of disease duration (<1 year to >13 years), but has limitations in terms of sociodemographic diversity. All were White, English speaking and educated, with over half retired/medically retired. The promotion of an intersectional approach that emphasises the intersectionality of PD with other contextual aspects such as ethnicity and socioeconomic status [79, 80] and disease characteristics (stages of disease progression [81], age at PD onset [82]) could offer additional insights into the impact of NMS. Moreover, those who took part likely had a general interest in NMS, compared to other persons with PD.

## 5 Conclusions

This photovoice study provided a unique opportunity to capture the far‐reaching impact of the NMS of PD on activities of daily living and QoL in real time. Participants experienced barriers to socialising, engaging in valued activities and going out in public places. Mental health disturbances and challenges to sense of self had a negative impact on well‐being and self‐esteem. However, implementing coping strategies and receiving social support helped buffer against these negative factors and enhanced QoL. Our findings demonstrate the importance of providing personalised psychological and occupational support for people with PD, focusing on engagement in valued activities and adopting a positive mindset. Targeted information provision to help empower patients and families to self‐manage is likely to be beneficial, as well as public awareness campaigns of the multifaceted symptoms of PD to reduce social stigma and facilitate engagement within society.

## Author Contributions

**Laura J. Smith:** conceptualisation, funding acquisition, writing–original draft, methodology, project administration, formal analysis, supervision, writing–review and editing, resources, investigation. **Jerri Callis:** writing–original draft, formal analysis, project administration, data curation, writing–review and editing, methodology. **Shannon Bridger‐Smart:** formal analysis, investigation. **Olivia Guilfoyle:** formal analysis, investigation.

## Ethics Statement

The study was approved by the University of Kent Psychology Ethics Committee (202216473327287620). Participants provided informed consent before participation and gave verbal consent at the beginning of each interview.

## Conflicts of Interest

The authors declare no conflicts of interest.

## Supporting information

Supporting information.

## Data Availability

The data that support the findings of this study are available on request from the corresponding author. The data are not publicly available due to privacy or ethical restrictions.
