# Supplementary material for: Experiences of Living With the Nonmotor Symptoms of Parkinson's Disease: A Photovoice Study
Source: Health Expect. 2024 Jun 25;27(3):e14124. doi: 10.1111/hex.14124 (PMC11199325; doi:10.1111/hex.14124)
Supplement: Supplementary file 1 — Supporting information. [file HEX-27-e14124-s001.docx]

**Supplementary Materials**

1. Reflection Template P2-3

2. Coding Scheme P4-5

3. Standards for Reporting Qualitative Research (SRQR) Checklist P6-8

**Understanding the Impact of Non-Motor Symptoms in Parkinson’s Disease
Reflection Template**

| **Participant ID number:** |
| --- |
|  |
| **Label:** *Give the photograph a ‘name’ or identifier. This can be one word or a few words. This will help us to link the photograph to your explanation.* |
|  |
| **What is happening in the picture?** *(Describe so others will know what the picture is of.)* |
|  |
| **What does it mean to you?** *(Why is it meaningful?)* |
|  |
| **Why is it important to you?** *(What does it mean to you, why is it relevant to you?)* |
|  |
| **How does it relate to your non-motor symptoms?** *(What symptoms does it represent?)* |
|  |
| **How does it relate to your ability to carry out daily activities?** *(How does it relate to your daily tasks and routines?)* |
|  |
| **How does it relate to your enjoyment of life and wellbeing?** *(What is the impact on your health and happiness, how does it make you feel?)* |
|  |
| **What can we learn from this? What can be done about this?** *(What would you like others to know?)* |
|  |
| **Any other reflections:** |
|  |

| **Code** | **Description** |
| --- | --- |
| **Symptomology** | |
| Sensory disturbances | Anosmia, ageusia, visual disturbances. |
| Fatigue and sleep | Feeling mentally/ physically tired or drained. Sleep disturbances. |
| Cognitive problems | Difficulty concentrating, remembering, communicating, etc. |
| Bladder and bowel function | Urge incontinence, nocturia, constipation. |
| Movement and balance | Slowness of movement, falls and dizziness, tremor, rigidity. |
| Unpredictability | Fluctuations day-to-day, symptoms appearing without warning. |
| Interacting symptoms | Symptoms interrelate and can exacerbate each other. |
| Other |  |
| **Daily activities** | |
| Opportunities/ accessibility | Given space to participate and engage, usable environments and services. |
| Ability to undertake activities | Ability to carry out daily tasks including work, chores, travel, and driving. |
| Barriers to undertaking activities | Perceived barriers to daily functioning including physical capability, environment, energy, motivation etc. |
| Impact on quality of life | Being able to complete daily activities positively impacts quality of life, whereas quality of life is poorer when activities cannot be undertaken. |
| Other |  |
| **Mental health** | |
| Embarrassment | Shame, embarrassment, negative self-evaluation, social reputation. |
| Anxiety | Worried, stressed, apprehensive, panic, avoidance. |
| Low mood | Unhappiness, hopelessness, apathy. |
| Grief | Sorrow, loss during the illness trajectory, life ‘taken’ by Parkinson’s disease. |
| Frustration | Irritability, anger, annoyance. |
| Other |  |
| **Identity** | |
| Normality | Maintaining normality to preserve sense of self. Keeping up with usual routines and activities. |
| Confidence | Loss of confidence and ways to maintain or enhance confidence. |
| Dependence | Dependency or reliance on others. |
| Independence and freedom | Retaining agency, independence, sense of control. |
| Self-esteem | Self-image, self-worth, self-compassion, self-stigma. |
| Purpose and achievement | Personal goals, finding sense of purpose and meaning, sense of mastery, hope. |
| Social roles | Withdrawing from accustomed social roles within the workplace, home, and relationships. |
| Other |  |
| **Interactions with the public** | |
| Perceptions and attitudes from others | Social stigma, assumptions made by others. |
| Non-visible or hidden symptoms | Symptoms that are difficult to observe from the outside are often neglected or misunderstood. |
| Need for awareness, understanding, and education | Community knowledge, approaches to reduce stigma and enhance understanding of non-motor symptoms, educational campaigns. |
| Need for behaviour change from others | Responding patiently and empathetically, adjustments, clear communication styles. |
| Other |  |
| **Relationships** | |
| Impact on loved ones | How relationships with friends and family are affected, changes in the dynamics of relationships. |
| Support from relationships | Emotional and practical support to enable participation and promote positive wellbeing. |
| Patience and empathy | Compassion, active listening, receptive to needs, validating concerns. |
| Connections and closeness | Connections or bonds between those close to us (family, partner, friends). |
| Companionship and peer support | Shared experiences, reciprocity, social connectedness, mutual understanding. |
| Isolation and social withdrawal | Loneliness, reduced contact with others, feeling excluded. |
| Other |  |
| **Coping strategies** | |
| Outdoors and nature | Being outside and experiencing nature, outdoor activities, and favourite outdoor places. |
| Exercise | Exercise classes, recreational sports, gym, physiotherapy. Being physically active is beneficial to mental and physical wellbeing. |
| Hobbies | Valued activities that provide enjoyment including arts, music, reading, travel, and games. |
| Planning and pacing | Planning daily activities ahead of time and spacing these appropriately allowing for rest. |
| Stress management | Techniques to promote relaxation and improve mood, including mindfulness, yoga, and Cognitive Behavioural Therapy. |
| Medication | Managing and controlling symptoms. Establishing a medication regime and dose timings. |
| Other |  |
| **Mindset** | |
| Adjusting to life with Parkinson’s disease | Behavioural and psychological adjustments implemented as part of an ongoing process. |
| Positivity | Focusing on good things and adopting an optimistic attitude. As opposed to rumination and dwelling on negatives. |
| Determination | Working through challenges and facing up to Parkinson’s disease. |
| Learning and keeping informed | Staying informed, following research updates, empowerment, making sense of experiences. |
| Preventative strategies to keep well | Lifestyle changes adopted to support healthy living for longer. Including diet, exercise, and cognitive games. |
| Other |  |

|  | **Standards for Reporting Qualitative Research (SRQR)*** |  |
| --- | --- | --- |
|  | <http://www.equator-network.org/reporting-guidelines/srqr/> |  |
|  |  | **Page/line no(s).** |
| **Title and abstract** | |  |
|  | **Title** - Concise description of the nature and topic of the study Identifying the study as qualitative or indicating the approach (e.g., ethnography, grounded theory) or data collection methods (e.g., interview, focus group) is recommended | P1 and title page |
|  | **Abstract** - Summary of key elements of the study using the abstract format of the intended publication; typically includes background, purpose, methods, results, and conclusions | P1 (Abstract) |
|  |  |  |
| **Introduction** | |  |
|  | **Problem formulation** - Description and significance of the problem/phenomenon studied; review of relevant theory and empirical work; problem statement | P2-3 (Introduction) |
|  | **Purpose or research questio**n - Purpose of the study and specific objectives or questions | P3 |
|  |  |  |
| **Methods** | |  |
|  | **Qualitative approach and research paradigm** - Qualitative approach (e.g., ethnography, grounded theory, case study, phenomenology, narrative research) and guiding theory if appropriate; identifying the research paradigm (e.g., postpositivist, constructivist/ interpretivist) is also recommended; rationale** | P3 (Design) |
|  | **Researcher characteristics and reflexivity** - Researchers’ characteristics that may influence the research, including personal attributes, qualifications/experience, relationship with participants, assumptions, and/or presuppositions; potential or actual interaction between researchers’ characteristics and the research questions, approach, methods, results, and/or transferability | P3 (Design) |
|  | **Context** - Setting/site and salient contextual factors; rationale** | P2 (Introduction) P4 (Participants) |
|  | **Sampling strategy** - How and why research participants, documents, or events were selected; criteria for deciding when no further sampling was necessary (e.g., sampling saturation); rationale** | P3-4 (Participants) P4 Table 1 |
|  | **Ethical issues pertaining to human subjects** - Documentation of approval by an appropriate ethics review board and participant consent, or explanation for lack thereof; other confidentiality and data security issues | P5 (Ethical considerations) |
|  | **Data collection methods** - Types of data collected; details of data collection procedures including (as appropriate) start and stop dates of data collection and analysis, iterative process, triangulation of sources/methods, and modification of procedures in response to evolving study findings; rationale** | P4-5 (Materials and procedure) |
|  | **Data collection instruments and technologies** - Description of instruments (e.g., interview guides, questionnaires) and devices (e.g., audio recorders) used for data collection; if/how the instrument(s) changed over the course of the study | P4-5 (Materials and procedure) |
|  | **Units of study** - Number and relevant characteristics of participants, documents, or events included in the study; level of participation (could be reported in results) | P6 (Results) |
|  | **Data processing** - Methods for processing data prior to and during analysis, including transcription, data entry, data management and security, verification of data integrity, data coding, and anonymization/de-identification of excerpts | P5 (Data analysis) |
|  | **Data analysis** - Process by which inferences, themes, etc., were identified and developed, including the researchers involved in data analysis; usually references a specific paradigm or approach; rationale** | P5-6 (Data analysis) |
|  | **Techniques to enhance trustworthiness** - Techniques to enhance trustworthiness and credibility of data analysis (e.g., member checking, audit trail, triangulation); rationale** | P3 (Design), P5-6 (Data analysis) |
|  |  |  |
| **Results/findings** | |  |
|  | **Synthesis and interpretation** - Main findings (e.g., interpretations, inferences, and themes); might include development of a theory or model, or integration with prior research or theory | P6-12 (Results) |
|  | **Links to empirical data** - Evidence (e.g., quotes, field notes, text excerpts, photographs) to substantiate analytic findings | P6-12 (Results) Figures 1-3 |
|  |  |  |
| **Discussion** | |  |
|  | **Integration with prior work, implications, transferability, and contribution(s) to the field -** Short summary of main findings; explanation of how findings and conclusions connect to, support, elaborate on, or challenge conclusions of earlier scholarship; discussion of scope of application/generalizability; identification of unique contribution(s) to scholarship in a discipline or field | P12-15 (Discussion) |
|  | **Limitations** - Trustworthiness and limitations of findings | P15 (Discussion) |
|  |  |  |
| **Other** | |  |
|  | **Conflicts of interest** - Potential sources of influence or perceived influence on study conduct and conclusions; how these were managed | Title page |
|  | **Funding** - Sources of funding and other support; role of funders in data collection, interpretation, and reporting | Title page |
|  |  |  |
|  | *The authors created the SRQR by searching the literature to identify guidelines, reporting standards, and critical appraisal criteria for qualitative research; reviewing the reference lists of retrieved sources; and contacting experts to gain feedback. The SRQR aims to improve the transparency of all aspects of qualitative research by providing clear standards for reporting qualitative research. |  |
|  |  |  |
|  | **The rationale should briefly discuss the justification for choosing that theory, approach, method, or technique rather than other options available, the assumptions and limitations implicit in those choices, and how those choices influence study conclusions and transferability. As appropriate, the rationale for several items might be discussed together. |  |
|  |  |  |
|  | **Reference:** |  |
|  | O'Brien BC, Harris IB, Beckman TJ, Reed DA, Cook DA. **Standards for reporting qualitative research: a synthesis of recommendations.** *Academic Medicine*, Vol. 89, No. 9 / Sept 2014  DOI: 10.1097/ACM.0000000000000388 |  |
|  |  |  |
|  |  |  |
